# Causes and consequences of intra-specific variation in vertebral number

**DOI:** 10.1038/srep26372

**Published:** 2016-05-23

**Authors:** Petter Tibblin, Hanna Berggren, Oscar Nordahl, Per Larsson, Anders Forsman

**Affiliations:** 1 Ecology and Evolution in Microbial model Systems, EEMiS, Department of Biology and Environmental Science, Linnaeus University, SE-391 82 Kalmar, Sweden

## Abstract

Intraspecific variation in vertebral number is taxonomically widespread. Much scientific attention has been directed towards understanding patterns of variation in vertebral number among individuals and between populations, particularly across large spatial scales and in structured environments. However, the relative role of genes, plasticity, selection, and drift as drivers of individual variation and population differentiation remains unknown for most systems. Here, we report on patterns, causes and consequences of variation in vertebral number among and within sympatric subpopulations of pike (*Esox lucius*). Vertebral number differed among subpopulations, and common garden experiments indicated that this reflected genetic differences. A *Q*_*ST*_-*F*_*ST*_ comparison suggested that population differences represented local adaptations driven by divergent selection. Associations with fitness traits further indicated that vertebral counts were influenced both by stabilizing and directional selection within populations. Overall, our study enhances the understanding of adaptive variation, which is critical for the maintenance of intraspecific diversity and species conservation.

The causes and consequences of discontinuous phenotypic variation have fascinated evolutionary biologists for more than a century^1^. Variation in number of vertebrae is potentially informative in this regard. The number of vertebrae varies extensively among and within species of organisms such as snakes^2^,^3^,^4^,^5^, legless lizards^6^, salamanders^7^ and fish^8^,^9^,^10^,^11^. Patterns of variation among species suggest that vertebral number (henceforth VN) is associated with ecology, life-style, body form (e.g., elongation), and body size^9^,^12^. For instance, comparative analyses of snakes have shown that VN depends on whether the species have a fossorial, terrestrial, arboreal or aquatic lifestyle, and differs between snakes with a constricting and non-constricting foraging mode^5^,^13^. Among species comparisons further indicate that the evolution of a larger and more elongated body shape has been accompanied by evolution of increasing VN (pleomerism) in both snakes^5^ and fish^11^,^14^.

Vertebral number varies also among populations within various species of fish, salamanders and reptiles^2^,^7^,^9^,^15^,^16^. Previous studies of intraspecific variation have aimed at evaluating whether VN increase with latitude in accordance with Jordan’s rule^17^,^18^,^19^, or report on comparisons in structured systems such as islands or lakes where populations are isolated^2^,^4^,^16^,^20^. Little is known about whether VN may vary also within less delineated systems or among sympatric populations due to local adaptations or plastic responses to population-specific reproductive habitats^15^,^21^.

Vertebrae segments are formed during early embryogenesis and the number of vertebrae is irreversible^18^,^22^,^23^,^24^. The evolution of an elongated snake- (and fish-) like body form with a de-regionalized pre-cloacal axial skeleton is best explained by retention of standard vertebrate *Hox* gene domains with alteration of downstream expression that suppresses development of distinct regions and increases somite numbers^25^,^26^,^27^. That intraspecific variation of VN has an additive and heritable genetic basis has been demonstrated by common garden experiments as well as by heritability estimates derived from quantitative genetics approaches^4^,^8^,^15^,^28^. Developmental plasticity in response to environmental factors, most notably temperature, salinity and light regime, can also contribute to variation in VN among individuals and populations^7^,^18^,^24^,^29^,^30^. However, few studies have investigated the relative roles of plasticity and genetic modifications as drivers of population divergence in VN^8^, and the degree to which genetically based differences (when present) reflect local adaptations in response to divergent selection rather than neutral processes has rarely been addressed^15^,^21^,^31^.

Several lines of evidence indicate that inter-individual variation in VN is associated with manoeuvrability and speed of locomotion^4^,^10^,^31^,^32^,^33^. Because it affects locomotion and agility, VN may influence susceptibility to predation and foraging performance, and ultimately contribute to variation among individuals in fitness^2^,^4^,^10^,^34^. Vertebral number may also influence the capacity for growth, because individuals with more vertebrae have more growth zones^2^,^35^. Observational and experimental evidence suggest that the positive effect of VN on growth capacity is expressed under favourable conditions that allow for rapid growth, but not under limited resources^36^. Previous studies have also reported on associations of VN with neonate body size^2^,^31^,^35^, juvenile growth rate^10^ and with survival^2^,^34^. However, to our knowledge, no studies have investigated whether and how individual variation in VN influences longer term growth trajectories and reproductive allocation strategies, although such differences can be hypothesized given differences in life expectancy.

Here, we report on an integrative study of patterns, causes, and consequences of variation in VN in six sympatric subpopulations of pike (*Esox lucius*). Pike is an iteroparous, long-lived and large keystone predatory fish species^37^, and an emerging model organism for studies in ecology and evolutionary biology^38^. The subpopulations included in this study share a sympatric forage habitat along a coastal area of the western Baltic Sea, and become allopatric only for a short period during reproduction and early life-stages in small streams due to migratory homing behaviour^39^,^40^,^41^. Subpopulations are genetically differentiated (as evidenced by analysis of microsatellite marker data)^40^ and display local adaptations of larval traits^42^ and in growth trajectories and adult body size to their specific breeding habitats^39^.

Pike have an elongated body and indeterminate growth^37^, use an ambush attacking strategy^43^, and display migratory behaviour^39^,^40^ -traits that may impose selection on VN. Pike also have structures making them amenable for indirect study and reconstruction of life-history. For instance, analysis of annual growth rings in the cleithrum^44^ allows for age determination and for reconstruction of growth trajectories at the level of individuals or populations^38^. Our study system thus provides interesting opportunities to investigate genetic and environmental sources of phenotypic variation, evolutionary divergence among populations, and to investigate current selection by examining associations of variation in VN with fitness components at the level of individuals within populations.

In this study, we first quantified and compared variation in VN among and within subpopulations of pike by sampling adults and juveniles (less than 6 weeks old) in six breeding streams (Fig. 1a–c). Next, we performed a common garden experiment to evaluate genetic and plastic components of variation in VN. Data from the common garden experiment were also used to estimate quantitative phenotypic differentiation (*Q*_*ST*_) and then combined with measures of neural genetic differentiation (*F*_*ST*_, based on microsatellite marker data) to assess whether divergence among subpopulations was adaptive by a *Q*_*ST*_-*F*_*ST*_ comparison^15^,^39^,^45^. Additionally, we conducted a split-brood experiment and reared families under different incubation temperatures to quantify the magnitude of phenotypic plasticity of VN in response to temperature during embryonic development, and to test for effects of genotype by environment (G × E) interactions. Finally, we used information from experimental and wild-caught fish to test whether variation among individuals in VN was associated with survival, growth rates, age-specific body size, or female reproductive effort.

## Results

### Comparisons of vertebral number among subpopulations

**Figure 1:**
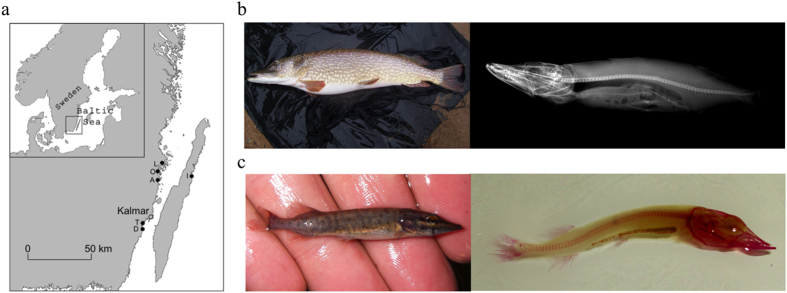
Study area and methods for visualising the vertebrae column. (**a**) Map of study area with geographic locations of breeding streams for sampled *Esox lucius* pike subpopulations. Note that individuals from different subpopulations coexist in the Baltic Sea during the majority of the life cycle. See Table 1 for further details of the locations, key to abbreviations and their contributions to the study. The map was generated in Adobe Photoshop CS5 Extended, version 12.0.4 × 32, http://www.adobe.com/se/products/photoshop.html. The image was modified from a base map available under non-restrictive creative commons license obtained from https://commons.wikimedia.org/wiki/File:Scandinavia-template.png (**b**) Vertebrae column of adult (ca. 90 cm long) pike visualised by x-ray. Photos by Petter Tibblin. (**c**) Vertebrae column of juvenile (ca. 5 cm) pike visualised by Alizarin Red staining. Photos by Oscar Nordahl.

**Figure 2:**
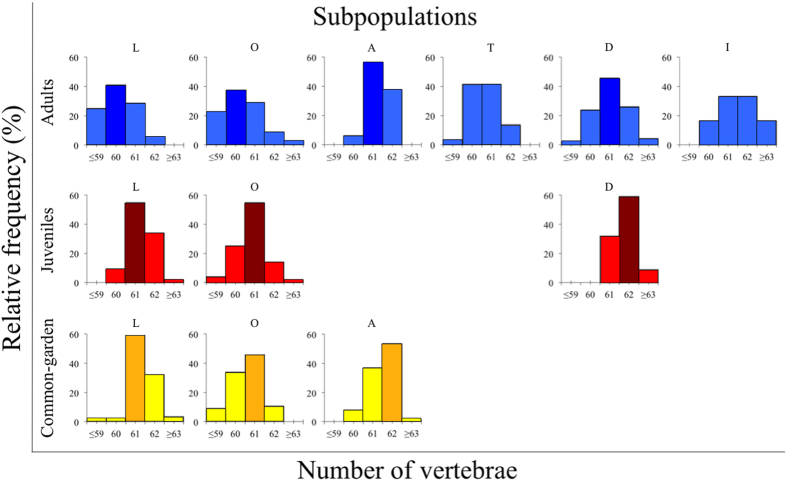
Variation in vertebral number among subpopulations and life-stages. Frequency distribution (%) of vertebral number in sympatric subpopulations of *Esox lucius* pike and throughout different life-stages (adults (blue), wild-caught juveniles (red) and common-garden reared (in a constant temperature of 14 °C) juveniles (yellow)). Darker coloured boxes illustrate the most frequent vertebral number in different subpopulations and life-stages. For a key to abbreviations of subpopulations (L, O, A, T, D, and I), see Table 1.

Data for wild-caught individuals showed that VN varied among subpopulations in both adults (Fisher’s Exact test: *χ*^2^ = 57.39, df = 15, *N* = 328, *P* < 0.0001) and juveniles (*χ*^2^ = 27.57, df = 6, *N* = 146, *P* < 0.0001, Fig. 2). Despite that breeding streams were only separated by short distances (Fig. 1a), the magnitude of the differences between population averages was large, corresponding to 18% (1.4/8, adults) and 14% (1.0/7, juveniles) of the total range of variation seen among individuals, and to 1.27 (1.4/1.1, adults) and 1.25 (1.0/0.8, juveniles) of the standard deviation of the pooled sample of individuals (Fig. 2).

### Variation in vertebral number among subpopulations accounted for by genetic modifications

A central question was whether differences among subpopulations in VN reflected genetic modifications, developmental plasticity or a combination of the two. This was evaluated using data for juveniles reared in the common garden experiment, the results of which uncovered significant differences in frequency distribution of VN among subpopulations (Fisher’s Exact test: *χ*^2^ = 56.41, df = 6, *P* < 0.0001, Fig. 2c). The magnitude of the differences between subpopulation averages in the common garden corresponded to 18% (0.9/5) of the total range of variation seen among individuals, and to 1.1 (0.9/0.8) of the standard deviation of the pooled sample of individuals (Fig. 2). This result indicated that differences were, at least in part, genetically determined.

### Resemblance between captive reared and wild-caught juveniles indicated that population differences reflected genetic modifications rather than plasticity

In two subpopulations (L & O) data allowed for comparisons of VN between wild-caught and captive-reared (common garden) juveniles to evaluate the hypothesis that differences between subpopulations represented plastic responses to environmental components. However, VN did not differ between juveniles that had developed in different environments in any of the subpopulations (subpopulation L: Fisher’s Exact test: *χ*^2^ = 3.97, df = 3, *P* = 0.22; subpopulation O: Fisher’s Exact test: *χ*^2^ = 3.15, df = 3, *P* = 0.38, Fig. 2). The range of VN in captive-reared juveniles (59–63) as well as the magnitude of the differences between subpopulations resembled those seen in wild-caught juveniles (Fig. 2). This similarity between wild-caught and common garden reared juveniles indicated that the variation among subpopulations seen in wild-caught individuals likely represented genetic modifications rather than developmental plasticity.

### Developmental plasticity of vertebral number in response to incubation temperature

**Figure 3:**
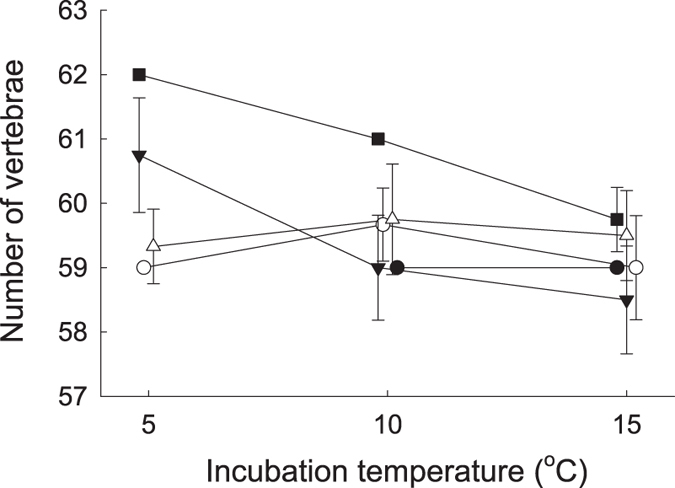
Developmental plasticity of vertebral number in response to incubation temperature. Figure shows genotype by environment interactions as indicated by within-family comparisons of vertebral number in *Esox lucius* pike reared under three temperature treatments. Figure shows means ± s.d. Symbols indicate data for five split-brood families originating from subpopulation O. Data on temperature have been jittered in the x-axis direction.

The split-brood experiment, in which full-siblings were exposed to different incubation temperatures (5,10, 15 and 20 °C), uncovered that VN decreased significantly with increasing temperatures (GLM, effect of treatment: *F*_2,36_ = 4.82, *P* = 0.014, effect size *η*^2^ = 0.09) and that there were significant effects of family (GLM, effect of family: *F*_4,36_ = 5.89, *P* = 0.001, *η*^2^ = 0.22) (Fig. 3). None of the eggs incubated in the 20 °C treatment hatched. The results from the split-brood experiment further uncovered crossing norms of reaction (as evidenced by a significant effect on vertebral number of the family by temperature interaction, GLM: *F*_7,36_ = 3.01, *P* = 0.014, *η*^2^ = 0.20, Fig. 3), suggesting that there existed genetic variation for plasticity.

### *Q*_*ST*_-*F*_*ST*_ comparison revealed that the divergence in vertebral number among subpopulations was driven by selection

The quantitative genetic differentiation (as estimated by *Q*_ST_) of VN was high (*Q*_*ST*_ = 0.54, 95% CI = 0.24–1.00) and it significantly exceeded the neutral genetic expectation set by *F*_*ST*_ (0.069, 95% CI = 0.048–0.085), as illustrated by non-overlapping 95% confidence intervals. Theory states that subpopulation divergence is due to divergent selection if *Q*_ST_ is significantly different from *F*_ST_^45^,^46^,^47^. Our results thus indicated that differences among subpopulations could be ascribed to divergent selection rather than to genetic drift.

### Differences in vertebral number between adults and juveniles indicated viability selection

Comparisons of VN frequency distributions revealed differences between wild-caught adults and juveniles, after controlling for the variation among subpopulations (Log-linear model; effect of life-stage: χ^2^ = 17.85, df = 1, *P* < 0.0001; effect of subpopulation: χ^2^ = 11.47, df = 2, *P* = 0.003). Adults had lower VN than juveniles in all three subpopulations (Fig. 2), suggesting that variability among individuals within populations was affected by natural (viability) selection.

### Association of vertebral number with growth rate in juveniles and subadult life-stages

**Figure 4:**
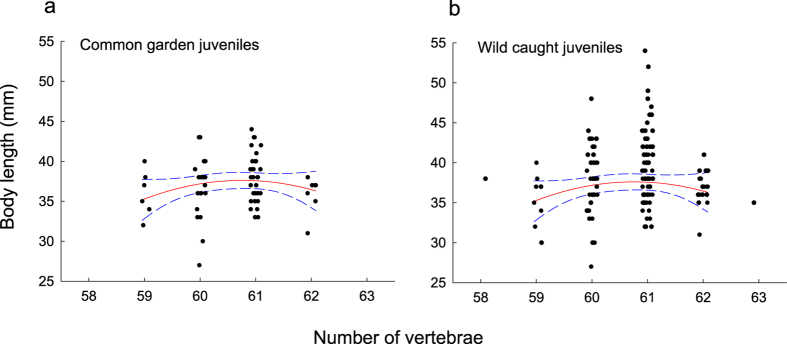
Relationship between juvenile body length and vertebral number. (**a**) Juveniles that were reared in a common garden experiment. (**b**) Juveniles that were captured in the wild upon leaving their breeding ground. All data are for *Esox lucius* pike individuals from subpopulation O. The relationship in wild caught juveniles remained significant when data for the two individuals with 58 and 63 vertebrae were omitted from the analysis (test of linear effect: *t* = 3.10, *P* = 0.0028, effect size *η*^2^ = 0.13; test of quadratic (curvilinear) effect: *t* = −3.09, *P* = 0.0029, effect size *η*^2^ = 0.13). Dashed blue lines indicate 95% CI. Data on vertebral number have been jittered in the x-axis direction.

Data on body size indicated stabilizing selection^48^ on VN in juveniles. Individuals with an intermediate number of vertebrae were larger on average compared with individuals having either relatively few or relatively many vertebrae (Fig. 4a,b). Quadratic regression demonstrated a statistically significant curvilinear relationship between body size and VN in wild-caught juveniles (y = −4183 + 139.1 (±57.6 SE) X–1.14 (±0.475 SE) X^2^; test of linear effect: *t* = 2.42, *P* = 0.0184, effect size *η*^2^ = 0.08; test of quadratic effect: *t* = −2.41, *P* = 0.0186, *η*^2^ = 0.08, randomization test for significant quadratic effect, *p* = 0.029 (28/2000)) but not in common garden reared juveniles (y = −2757 + 92.1 (±60.93) X–0.75 (±0.50) X^2^; test of linear effect: *t* = 1.51, *P* = 0.136, effect size *η*^2^ = 0.03; test of quadratic effect: *t* = −1.50, *P* = 0.138, *η*^2^ = 0.03, randomization test for significant quadratic effect, *p* = 0.26 (259/2000)). These results indicated that the relationship linking body length to VN was expressed more strongly among individuals under natural conditions in the wild than in the laboratory (Fig. 4a,b). However, the shape of the relationship did not differ between captive-reared and wild-caught juveniles (effect of interaction between origin and the linear effect: *F*_1,130_ = 0.27, *P* = 0.61, *η*^2^ = 0.0017; effect of interaction between origin and the (squared) curvilinear effect: *F*_1,130_ = 0.26, *P* = 0.61, *η*^2^ = 0.0017).

**Figure 5:**
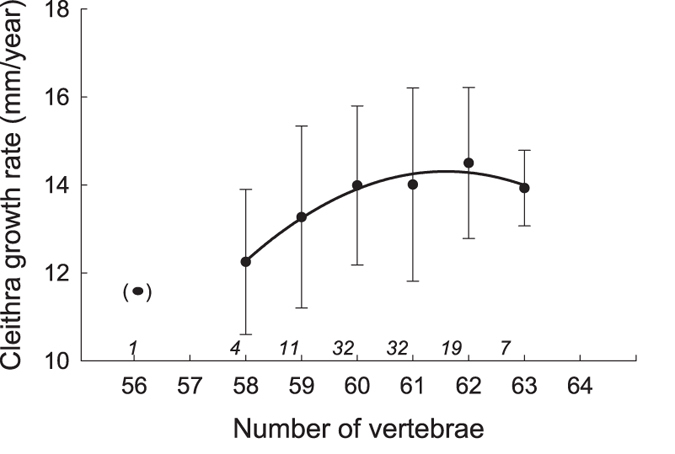
Relationship between individual growth rate and vertebral number. Growth rate of *Esox lucius* pike as reconstructed based on measurements of the first three annual growth zones in cleithrum and representing length increment (per year) during the first three years of life. Numbers in italics above the horizontal axis denote sample sizes. The curvilinear relationship between growth rate and vertebral number remained significant when data for the outlier individual with 56 vertebrae was omitted from the analysis (quadratic regression analysis based on mean values for each vertebral number class, y = −586.6 + 19.5 (±3.93 SE) X–0.16 (±0.033 SE) X^2^; test of linear effect: *t* = 4.96, *P* = 0.0157; test of quadratic effect: *t* = −4.87, *P* = 0.0165, the full model was significant *F*_2,3_ = 38.4, *P* = 0.0073, *R*^2^ = 0.96). Figure panel show means ± s.d. Data are for individuals from subpopulation O.

Comparisons of reconstructed annual growth rates (based on zonation of the cleithra bone^39^,^44^) during the first three years of life (*i.e.* the sub-adult life-stage) among wild-caught individuals with different numbers of vertebrae uncovered a curvilinear relationship, indicative of stabilizing selection (Fig. 5). Quadratic regression analysis confirmed that the curvilinear relationship between individual growth rate of cleithrum and VN was statistically significant (analysis based on mean values for each vertebral number class, y = −480.1 + 16.0 (±1.84 SE) X–0.13 (±0.015 SE) X^2^; test of linear effect: *t* = 8.72, *P* = 0.0010, *η*^2^ = 0.21; test of quadratic effect: *t* = −8.40, *P* = 0.0011, *η*^2^ = 0.19; the full model was significant *F*_2,4_ = 182.5, *P* < 0.0001, *R*^2^ = 0.99). The signature of stabilizing selection during the sub-adult life-stage was similar to that seen in juveniles (*cf*. Figs 4 and 5).

### Association of vertebral number with female reproductive effort

**Figure 6:**
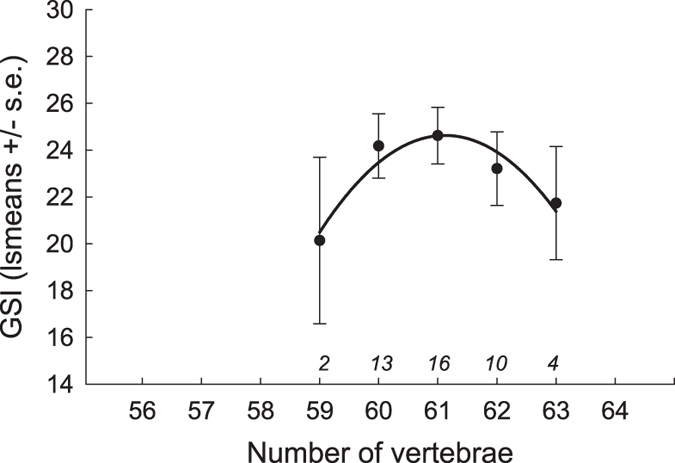
Relationship between reproductive investment and vertebral number in females. Figure shows least square means ± s.e. of Gonad Somatic Index (GSI). See text for details. Data are for *Esox lucius* pike individuals from subpopulation O. Numbers in italics above the horizontal axis denote sample sizes.

Comparisons of reproductive effort, as estimated using Gonad Somatic Index^49^, among individuals with different numbers of vertebrae uncovered a curvilinear relationship indicative of stabilizing selection (quadratic regression analysis based on least squares means values for each VN class, y = −3409.6 + 112.38 (±25.81 SE) X–0.92 (±0.21 SE) X^2^; test of linear effect: *t* = 4.35, *P* = 0.0489, effect size *η*^2^ = 0.84; test of quadratic effect: *t* = −4.35, *P* = 0.0491, *η*^2^ = 0.83, Fig. 6).

## Discussion

We examined causes and consequences of variation in VN among and within six sympatric anadromous subpopulations of *E. lucius* pike using data for 721 wild-caught and captive-reared individuals. Our main findings were: *i*) number of vertebrae varied among subpopulations in the natural environment, *ii*) variation among subpopulations was, at least in part, genetically based and driven by divergent stage-specific selection as evidenced by a common garden experiment and a *Q*_*ST*_-*F*_*ST*_ comparison, *iii*) resemblance between captive-reared and wild-caught individuals suggested that the pattern of variation documented in natural subpopulations reflected genetic differences rather than plasticity, *iv*) cross-sectional comparisons indicated directional viability selection on VN, *v*) associations with juvenile body size, growth trajectories and reproductive effort suggested that individuals with an intermediate number of vertebrae had higher fitness, indicative of stabilizing selection within subpopulations.

Population divergence in VN has typically been evaluated across large geographic distances, commonly to assess whether patterns of variation conform to Jordan’s rule^17^,^19^. Considerably less attention has been directed towards documenting and understanding patterns of variation among populations within less delineated systems at small spatial scales^2^,^15^,^21^. In this study, we demonstrated that the frequency distribution of VN varied among pike that were sympatric in the Baltic Sea but originated from different subpopulations that utilize specific breeding streams due to homing behaviour^39^. The magnitude of the differences between subpopulation averages documented in this study (which corresponded to 1.26 s.d.) was large compared to previous, albeit few, studies of divergence in VN in confined systems and sympatric populations. For example, Manier, *et al*.^15^ found that divergence among six local populations of the Garter snake (*Thamnophis elegans*) corresponded to 0.5 s.d., whereas Aguirre, *et al*.^9^ found that two partially sympatric populations (anadromous and resident) of three-spine stickleback (*Gasterosteus aculetus*) were differentiated by 0.09 s.d.

Phenotypic variation can represent underlying genetic differences, environmentally induced plasticity, or reflect a combination of the two^50^,^51^,^52^. Several studies have shown that genetic components are involved in intraspecific variation of VN and that heritability is relatively high^2^,^4^,^8^,^28^. The results from our common garden experiment indicated that variation in VN among subpopulations was, at least in part, genetically based. Because we used only a F1-generation non-genetic maternal effects could also have been involved^4^,^8^. However, we produced common garden juveniles by stripping gametes from adults living in a shared environment prior to reproduction^39^. The influence of any environmentally induced (maternal effects) variation should therefore have been negligible.

Few studies have evaluated the relative roles of genetic components and plasticity in shaping variation in VN among populations^2^,^7^,^8^. It is well established that incubation temperature during embryogenesis can induce variation in VN^18^,^24^. To evaluate the role of temperature-induced plasticity we manipulated the incubation temperature in a split-brood experiment. Admittedly, the number of families in the split-brood experiment was small. Despite this, results uncovered that VN decreased with increasing temperature, which is coherent with most prevailing empirical evidence in other species^4^,^24^. Moreover, this experiment revealed genotype by environment effects on VN, emphasizing that there existed genetic variation of reaction norms allowing for evolution of developmental plasticity^53^. While it is possible that temperature-induced plasticity could have contributed to the differences in VN among subpopulations documented in wild-caught individuals, this would have required considerable and unrealistic temperature differences (approximately ∼10 °C, compare Figs 2 and 3) between breeding habitats. Any such temperature differences between breeding habitats should have been manifest as a disparity in VN between wild-caught and captive-reared juveniles. However, results for wild-caught and captive-reared juveniles were similar, suggesting that subpopulation differences reflected genetic effects rather than plastic responses to different breeding environments.

Population divergence in VN has been described in other species but less explored is whether such divergence is adaptive^15^,^21^. We addressed this by comparing phenotypic divergence (*Q*_*ST*_) in the common garden experiment with divergence at neutral microsatellite loci (*F*_*ST*_) and found that evolutionary divergence among subpopulations could be better explained by divergent selection than by random genetic drift. The *Q*_*ST*_-*F*_*ST*_ comparison included only three of the six populations. The results nevertheless indicated that differences in VN among these three subpopulations were shaped by local adaptations to their specific breeding streams. That subpopulations were subjected to different environments only for a fraction of the life-cycle^39^ suggests that phenotypic divergent selection during allopatry was intense^11^,^54^. This finding adds to the knowledge and understanding of adaptive variation in VN among fish populations, a phenomenon that previously was documented mainly for the Atlantic Silverside (*Menida menida*), for example by Hice, *et al*.^21^.

To identify the underlying selective driver(s) for the documented divergence would have required a different set of approaches than the ones used in the present study, but we offer two plausible explanations related to the association between VN and swimming performance. Performance studies indicate that VN is associated with locomotion and manoeuvrability^4^,^31^,^33^,^55^, for instance, by influencing body flexibility and the ability to curve the body^56^,^57^. Moreover, stellar work on sticklebacks proposes that VN mediated differences in swimming performance can translate into differential predation, particularly in juvenile life-stages, such that an optimal vertebral phenotype is favoured^10^,^34^,^58^. It is therefore reasonable to hypothesize that dissimilar predation pressures in population-specific breeding habitats may have resulted in divergent selection on VN in pike juveniles. This may ultimately have contributed to subpopulation divergence, in a similar manner as previously suggested for adaptive divergence in growth trajectories of pike^39^. Another explanation is that the subpopulation divergence in VN may have arisen due to the endeavours of the migratory life-history of pike. Given that VN influences swimming performance (as discussed above) it is plausible that VN can also influence migratory success and the ability to cope with physical obstacles faced during migration^33^. The variation in VN among subpopulations may therefore in part reflect differences in physical obstacles, for example rapids, which must be passed to reach breeding sites^55^.

The adaptive significance of individual variation in VN has been emphasized by studies reporting on associations between vertebral phenotypes and fitness correlates^2^,^4^,^34^,^58^,^59^. Much focus has been on whether VN is positively associated with body size^11^,^20^,^59^. We found a curvilinear relationship between VN and body size in juveniles indicating that an intermediate trait value was optimal, similarly to what was shown by Arnold^4^ in the garter snake. This pattern might reflect that locomotor performance peaks at intermediate number of vertebrae and that this translates into higher foraging success^10^. Our data further revealed that the relationship between VN and body size was expressed more strongly in the natural environment than in the laboratory, as previously shown in snakes^36^. A reasonable explanation for such context dependence is that any differences between vertebral phenotypes in performance (mobility) would likely be of higher value in a natural more challenging environment, where individuals need to capture free-moving prey, than in the laboratory. An additional explanation is that the inferior performance of individuals with extreme VN might partly reflect correlated responses to environmental stress (see below), and that any malfunctions associated with perturbed developmental processes manifested more strongly in the wild.

Related to the context dependent effects of VN on body size discussed above is the suggestion that the influence of phenotypic trait values on fitness can vary depending on sex, size class or age^3^,^4^,^7^,^34^. Still, few studies have evaluated whether effects of variation in VN on body size persist through time. By reconstructing growth trajectories using cleithra, we demonstrated that the curvilinear relationship between VN and body size documented in juveniles was present at least until individuals reached maturity. It has been suggested, but not empirically tested, that variation in VN also can influence reproductive allocation strategies independently of any effects of VN on body size^9^,^11^,^24^,^34^,^60^. We tested this hypothesis and found that female reproductive effort adjusted for body size (GSI) reached a peak in individuals with an intermediate number of vertebrae. It remains to be investigated whether this applies also to male reproductive investment.

The signatures of stabilizing selection on body size, growth rate and reproductive investment discussed above might reflect a combination of the aforementioned direct effect of VN on mobility and performance^4^,^10^,^31^,^32^,^33^ and indirect effects stemming from influences of environmental stress on gene expression and developmental processes^61^. Available evidence indicates that relatively high and relatively low VN are associated with exposure to extreme temperature conditions during early embryonic development^4^,^18^,^24^. If extreme temperatures modify gene regulation, activate otherwise unexpressed genes, and impair canalization this can induce developmental perturbations and lead to fitness reducing phenotypic abnormalities^61^ that may be related to but not caused by VN as such.

Given the associations with mobility and body size (growth rate), VN is likely subjected to viability selection^4^,^10^. We compared juvenile and adult life-stages and found that VN decreased across life-stages in all three subpopulations, indicative of directional viability selection. The duration of sampling ranged between 1 and 4 years depending on population and life-stage, and our samples of adults included individuals of different age (∼3–10 years). It is therefore unlikely that extreme environmental conditions during certain year(s) systematically affected VN distributions, or that the comparisons of different cohorts confounded environmental influences on vertebral development with selection. Our result also is congruent with previous studies on three-spine sticklebacks^10^,^58^,^60^.

Taken together, our longitudinal and cross-sectional analyses of associations between VN and different fitness components suggested that the variation in VN within populations is influenced by a combination of stabilizing and directional selection. That the mode of selection differs among fitness components is common^34^,^62^,^63^,^64^, and may reflect trade-offs among different components of fitness, such that trait values that enhance survival may impair reproductive effort or growth^3^,^34^,^50^,^65^,^66^. Moreover, previous studies (e.g.^34^,^67^) show that the optimal VN phenotype shifts with changes in body size and/or age. This would result in stabilising selection within life-stages (i.e. adult or juvenile) but directional selection between life-stages, in agreement with our findings.

In conclusion, results showed that differences in VN in pike reflected a combination of genetic and environmental (temperature) effects, and influenced aspects of individual performance (body size, growth rate, reproductive effort and survival) that likely contribute to variation in lifetime reproductive success. The overall patterns could be accounted for by a combination of divergent selection contributing to differentiation among subpopulations, and stabilizing selection reducing variation within subpopulations. Future studies should investigate the contribution of any correlated and indirect effects associated with perturbed development to patterns of variation in VN and fitness components in fish and other organisms. It is noteworthy that signatures of adaptive divergence (which were of relatively large magnitude compared to previous studies conducted within similar spatial scales) were evident despite that subpopulations were only separated by short distances relative to dispersal capacity for a brief period during reproduction and early development^39^. Collectively, our findings expand the present understanding of adaptive variation at fine spatiotemporal scales. In an era of accelerating anthropogenic influence on ecosystems such knowledge is key to successful conservation and management of biodiversity and ecosystem functioning.

## Methods

### Study system and sampling procedures

Pike is a suggested keystone top-predator fish that occupies a wide variety of aquatic habitats in the northern hemisphere^37^. Typically pike forage using an ambush strategy^43^ and it is prone to attack prey that can be larger than half their own body length^37^. Pike has indeterminate growth and breeds yearly after reaching maturity at age 1–4^37^. In some systems, for example the Baltic Sea, pike undertake breeding migrations in excess of 10 km to reach suitable breeding habitats and reproduction occurs in shallow waters during spring (March-May) at water temperatures between 6 and 14 °C^37^,^41^,^68^.

For this study, we used six subpopulations of pike breeding in streams entering the Baltic Sea (salinity is around 0.7% in the coastal area) within a confined geographical area with a radius of 25 km (Fig. 1a; Table 1). These streams are known as reproductive and nursery habitats for genetically differentiated subpopulations of anadromous pike^40^. Lerviksbäcken (L), Oknebäcken (O), Törnebybäcken (T) and Dunöbäcken (D) were small streams (width of 1–3 m, average water flows less than 0,5 m^3^/s) whereas Alsterån (A) was a larger watercourse (width 20–30 m, average water flow 10 m^3^/s) and they all flowed through woodland and agricultural areas of the Swedish mainland. Kårehamnsbäcken (I) was equally small as streams L, O, T and D but flowed through the agricultural landscape of the island of Öland (Fig. 1a). Marking studies to monitor timing and direction of migration have indicated that the majority of adult pike originating from these streams have a migratory life-history where they only entered the streams during breeding periods and subsequently emigrated back to the Baltic Sea within a few weeks^40^,^41^,^69^.

We sampled juveniles and adults in breeding streams to study patterns, causes and consequences of variation in the number of vertebrae, both within and among subpopulations. Adults (*N* = 328) were captured using fyke-nets placed in the streams during breeding migration (March-May). Individuals were measured for total body length (to the closest cm), from tip of snout to end of caudal fin, using a measuring board. Juveniles (less than six weeks old) were captured (*N* = 126, Table 1) during emigration from the streams to the Baltic Sea using either hand-trawl or larval traps. Juveniles were measured for total body length using digital callipers (to the closest mm).

### Methods for counting the number of vertebrae

We counted the number of vertebrae in 721 (328 adults and 393 juveniles) individuals originating from six subpopulations and representing both wild-caught and laboratory reared individuals (Fig. 1a; Table 1). In adults, we counted the vertebrae by analysing radiographs (generated by the x-ray system Arcoma PanoRad, Mediel accessed at the County Hospital of Kalmar, Sweden) in the software program ImageJ and/or by dissection after all soft tissues were removed by boiling (Fig. 1b). In juveniles, vertebrae were visualized by staining the vertebral column using Alizarin red S (Acros Organics, New jersey, USA) according to a method described by Connolly and Yelick^70^ followed by dissection. Specimens were then photographed in a dissection microscope (Olympus Digital camera UC 30, Olympus Microscope SZX12) and vertebrae were counted by image analysis using ImageJ software (Fig. 1c). To determine the repeatability of VN counts, 69 individuals (*N*_adults_ = 39, *N*_juveniles_ = 30) were counted twice and blind with respect to the first count. Measurement repeatability (intraclass correlation coefficient)^71^ was very high for both life stages (adults: 99.1%, *F*_38,39_ = 114.16, *P* < 0.001; juveniles: 96.7%, *F*_29,30_ = 31.71, *P* < 0.001), with less than 1% and 4% of the variance in VN being due to measurement error in adults and juveniles, respectively.

### Quantifying patterns of variation among subpopulations in the natural environment

Patterns of variation in VN were examined by comparing the frequency distributions among subpopulations based on wild-caught individuals. Number of vertebrae ranged between 56 and 64 (range for each subpopulation: L: 58–64, O: 56–63, A: 60–62, T: 59–62, D: 58–63, I: 60–63, Fig. 3). Data were analysed by Fisher’s Exact test implemented in SPSS 22, and performed separately for adults and juveniles. This was done to avoid any confounding effects of differences in VN between life-stages (see Results). To avoid cells with small expected values, data of vertebral number were grouped as ≤59, 60, 61, or ≥62^34^.

### Disentangling sources of variation among subpopulations

To address whether genetic modifications accounted for variation in VN among subpopulations, we compared the frequency distribution of VN among juveniles reared in a common garden experiment. For detailed methods of this section see^39^. In short, we dry-stripped gametes from spermiating males (*N* = 35) and ovulating females (*N* = 33) that were captured (within 11 days to reduce any effects of reproductive timing) during the breeding migration in three streams (subpopulations L, O & A; Table 1). Gametes were put on ice and transported to the laboratory where gametes were dry-mixed and artificially fertilized by adding water originating from the experimental facility. Temperature (14 °C) and light regime (14L/10D) were held constant throughout the experiment.

To estimate the contribution of additive genetic variation to differences in VN, we used a maternal half-sib breeding design where each female was crossed with two males. Each unique male-female crossing contributed with five aquaria replicates containing five larvae each. Fertilized eggs hatched after 5–6 days of incubation, and offspring were then reared for 35 days and fed brine shrimp (*Artemia salina*) and cyprinid larvae (*Cyprinius* sp.) *ad lib* before the experiment was terminated and juveniles were measured for total body length (TL, mm). For this study, we counted VN in a random subsample of individuals produced in the common garden experiment (one aquaria replicate from each parental crossing, *N* = 203 juveniles).

To analyse whether frequency distributions of VN differed among subpopulations when reared in a common garden, we used Fisher’s Exact tests due to low expected frequencies and data of VN were grouped as presented above. We also compared the frequency distribution of VN in the common garden experiment to that in wild-caught juveniles to infer the plastic component by performing Fisher’s Exact test separately for each subpopulation. Analyses were performed in SPSS 22.

### Testing for developmental plasticity of vertebral number in response to temperature during embryonic development

To examine whether the variation in VN among individuals and subpopulations could be accounted for by differences in temperatures experienced during early embryonic development we performed a split-brood laboratory experiment and incubated eggs under four different temperature regimes (5, 10, 15 and 20 °C). This experiment was designed to also test for gene by environment interaction effects (G × E) on VN, indicative of genetic variation in reaction norms required for evolution of phenotypic plasticity. The experiment was conducted by first dry-stripping gametes from five females and five males originating from subpopulation O (Table 1). Gametes were crossed to obtain five full-sib family batches. Family egg batches were artificially fertilized (conducted according to the procedure described for the common garden experiment), split into four parts consisting of ca. 20 eggs each, and incubated in 2L aquaria in four temperature treatments with each family being represented in each temperature. After hatching, larvae fed on the yolk-sac until depletion and were thereafter provided brine shrimp (*Artemia salina*) *ad lib*. Individuals were reared until they reached a length of more than 25 mm before termination to facilitate counting the number of vertebrae after staining with Alizarin red S and dissection. The experimental temperature gradient (5–20 °C) was chosen to comprise the general range of breeding temperatures for pike^37^,^68^. Data on VN were analysed using procedure GLM in SPSS 22 by including temperature treatment, family and the treatment by family interaction as explanatory fixed factors.

### Evaluation of whether subpopulation divergence in vertebral number is adaptive by *Q*_*ST*_-*F*_*ST*_ comparison

To assess whether divergence among subpopulations in VN could be more readily accounted for by differences in selection pressures than by random genetic drift, we compared phenotypic differentiation from the common garden experiment (*Q*_*ST*_) with genetic differentiation at neutral microsatellite loci (*F*_*ST*_). For this comparison we used data for populations L, O and A (Table 1). *Q*_*ST*_ was estimated according to the method described by Spitze^46^ (*i.e. Q*_ST_ = σ^2^_b_/(2σ^2^_w_ + σ^2^_b_), where σ^2^_b_ and σ^2^_w_ are additive genetic variances between and within subpopulations^47^). Restricted maximum likelihood (REML) estimates of σ^2^_b_ and σ^2^_w_ were derived from a Linear Mixed model applied to the common garden data using procedure MIXED in SPSS 22. In this model, VN was set as the dependent variable, subpopulation as fixed factor, and female and male as random factors nested within subpopulation. To avoid confounding effects of non-genetic variance, only variance components based on males were used to estimate *Q*_*ST*_. The 95% CIs for *Q*_ST_ were estimated using the direct simulation data method^72^. The estimated neutral genetic differentiation (*F*_*ST*_) among subpopulations used in the common garden experiment was 0.069 (Bootstrapped 95% confidence interval = 0.048–0.085) as presented by Tibblin, *et al*.^39^.

### Testing for associations of variation in vertebral number with fitness components

We examined whether VN was associated with survival by a cross-sectional comparison of the frequency distribution of VN between wild-caught adults and juveniles representing three subpopulations (L, O & D, Table 1). Data were analysed using a Log-linear model as implemented in procedure GENLIN in SPSS 22 to be able to control statistically for any variation in vertebral number among subpopulations. Vertebral number (grouped as ≤59, 60, 61, or ≥62) was treated as dependent variable with a Poisson distribution, and life-stage (*i.e.* adults or juveniles), subpopulation and the life-stage by subpopulation interaction as explanatory factors. The likelihood ratio was set as method for the Chi Square statistics. The interaction was non-significant (*P* = 0.30) and subsequently deleted which resulted in a final model without interaction.

We evaluated whether and how variation among individuals in VN was associated with juvenile growth rate by analysing the relationship between body size (mm length) and VN in juveniles that had been reared in captivity (from the common garden experiment described above), and in juveniles that were captured in the wild upon leaving their breeding stream. To allow for comparisons between VN and different fitness components (see below), we used data only for juveniles originating from subpopulation O, which is the population with the largest sample size. Data were analysed using multiple regression as implemented with procedure REG in SAS. Body size was set as response variable, and VN and squared VN was included in the model to test for linear and curvilinear (quadratic regression) relationships with body size^48^. We first analysed captive-reared and wild-caught juveniles separately. Next, we evaluated the hypothesis that effects of VN on performance and growth might be context dependent and expressed more strongly in more challenging environments where manoeuvrability is more important. To this end, we analysed data for captive-reared and wild-caught juveniles simultaneously and tested for effects on body size of the interaction between origin and VN using procedure GLM in SAS. To further evaluate the evidence for a curvilinear effect we performed a randomization test procedure for quadratic regression. The observed values of body size were randomly reallocated to the VN values, a 2-tailed test for a non-zero quadratic effect was conducted, and the outcome of 2000 randomizations was used to compute an overall significance level using the SAS macro RTEST^73^.

To evaluate whether and how VN influenced individual growth rates throughout the first three years of life (sub-adult life-stage) we reconstructed growth trajectories by measuring annual growth rings (zonation patterns) in the cleithrum for a subsample of adults originating from subpopulation O (*N* = 108). We restricted the analyses to the first three growth rings to minimize confounding effects of any differences in reproductive strategies. In pike, length of cleithrum is linearly related to total body length and constitutes a reliable proxy of growth rate (for details see^39^). To test for associations between individual growth trajectories and VN we first calculated annual growth rate for each individual, computed average growth rate for all individuals that had the same VN, and then performed a multiple regression analysis based on the mean values to test for linear and curvilinear (quadratic) effects.

To evaluate whether and how VN influenced reproductive investment, we first calculated GSI (Gonad Somatic Index) for a subsample of individuals originating from subpopulation O (*N* = 48, Table 1). We sampled individuals when they were entering the spawning areas and only used ripe but not ovulating females. We are therefore confident that we sampled individuals at the peak of their GSI^37^. To calculate GSI we divided gonad wet mass with somatic body mass (measured with 10 g accuracy, Digital Scale E15302, Fox International Group Limited)^49^. Next, we estimated least squares means of GSI for all individuals that had the same VN. These were obtained from an ANCOVA model in which GSI was treated as the dependent variable, female body length was treated as a covariate, and vertebral number and year were treated as class variables (the model was significant: *F*_9,37_ = 2.28, *P* = 0.038). We then performed a multiple regression analysis based on the least square means to test for linear and curvilinear (quadratic) effects^4^,^31^.

### Ethics statement

Ethical approval for the study was granted by the Ethical Committee on Animal Research in Linköping, Sweden (approval 9-06 & 39-10). The methods were carried out in accordance with the approved guidelines.

## Additional Information

**How to cite this article**: Tibblin, P. *et al*. Causes and consequences of intra-specific variation in vertebral number. *Sci. Rep.* **6**, 26372; doi: 10.1038/srep26372 (2016).

## Figures and Tables

**Table 1:** Data of sampled streams (subpopulations), sampling years and sample sizes used in the different subprojects to study patterns, causes and consequences of variation in vertebral number in *Esox lucius* pike.

Stream	Lat. Long. Coordinates	Subprojects	Year	*N*
Lerviksbäcken (L)	N 57° 04.400′	Adults	2012–2013	56
	E 16° 31.100′	Juveniles, wild	2014	53
		Juveniles, common garden	2012	59
Oknebäcken (O)	N 57° 01.200′	Adults, Growth, GSI	2011–2014	169
	E 16° 26.700′	Juveniles, wild	2014	71
		Juveniles, common garden	2012	65
		Temperature experiment	2012	50
Alsterån (A)	N 56° 55.400′	Adults	2012–2013	16
	E 16° 25.900′	Juveniles, common garden	2012	73
Törnebybäcken (T)	N 56° 39.700′	Adults	2010	29
	E 16° 17.560′			
Dunöbäcken (D)	N 56° 38.200′	Adults	2013–2014	46
	E 16° 14.800′	Juveniles, wild	2014	22
Kårehamnsbäcken (I)	N 56° 57.500′	Adults	2014	12
	E 16° 53.000′			

Juveniles (wild-caught and common garden reared) refer to individuals younger than six weeks and adults are older than two years.

## References

[b1] Bateson W. Materials for the Study of Variation. (Macmillan, 1894).

[b2] Lindell L. E., Forsman A. & Merilä J. Variation in number of ventral scales in snakes: Effects on body size, growth rate and survival in the adder. Vipera berus. J. Zool. 230, 101–115 (1993).

[b3] Shine R. Vertebral numbers in male and female snakes: the roles of natural, sexual and fecundity selection. J. Evol. Biol. 13, 455–465, doi: 10.1046/j.1420-9101.2000.00181.x (2000).

[b4] Arnold S. J. Quantitative genetics and selection in natural population: Microevolution of vertebral numbers in the garter snake *Thamnophis elegans*. In Proceedings of the second international conference on quantitative gentics, Sunderland, Massachusetts. (eds Weir B. S., Eisen E. J., Goodman M. J., & Namkoong G.) 619–636 (Sinauer Associates, 1988).

[b5] Lindell L. E. The evolution of vertebral number and body size in snakes. Funct. Ecol. 8, 708–719, doi: 10.2307/2390230 (1994).

[b6] Greer A. E. The Biology and Evolution of Australian Lizards. (Surrey Beatty & Sons, 1989).

[b7] Jockusch E. L. Geographic variation and phenotypic plasticity of number of trunk vertebrae in slender salamanders, batrachoseps (Caudata: Plethodontidae). Evolution 51, 1966–1982, doi: 10.2307/2411017 (1997). 28565126

[b8] Alho J. S., Leinonen T. & Merilä J. Inheritance of vertebral number in the three-spined stickleback (*Gasterosteus aculeatus*). PLoS ONE 6, e19579, doi: 10.1371/journal.pone.0019579 (2011). 21603609 PMC3095613

[b9] Aguirre W. E., Walker K. & Gideon S. Tinkering with the axial skeleton: vertebral number variation in ecologically divergent threespine stickleback populations. Biol. J. Linn. Soc. 113, 204–219, doi: 10.1111/bij.12316 (2014).

[b10] Swain D. P. The functional basis of natural-selection for vertebral traits of larvae in the stickleback *Gasterosteus aculeatus*. Evolution 46, 987–997, doi: 10.2307/2409751 (1992). 28564394

[b11] Lindsey C. C. Pleomerism, the widespread tendency among related fish species for vertebral number to be correlated with maximum body length. J. Fish. Res. Board. Can. 32 (1975).

[b12] Galis F. . Fast running restricts evolutionary change of the vertebral column in mammals. Proc. Natl. Acad. Sci. USA 111, 11401–11406, doi: 10.1073/pnas.1401392111 (2014). 25024205 PMC4128151

[b13] Hampton P. M. Ventral and sub-caudal scale counts are associated with macrohabitat use and tail specialization in viperid snakes. Evol. Ecol. 25, 531–546 (2011).

[b14] Maxwell E. & Wilson L. Regionalization of the axial skeleton in the ‘ambush predator’ guild-are there developmental rules underlying body shape evolution in ray-finned fishes? BMC Evol. Biol. 13, 265 (2013). 24314064 10.1186/1471-2148-13-265 PMC3867419

[b15] Manier M. K., Seyler C. M. & Arnold S. J. Adaptive divergence within and between ecotypes of the terrestrial garter snake, *Thamnophis elegans*, assessed with F-ST-Q(ST) comparisons. J. Evol. Biol. 20, 1705–1719, doi: 10.1111/j.1420-9101.2007.01401.x (2007). 17714288

[b16] Berner D., Moser D., Roesti M., Buescher H. & Salzburger W. Genetic architecture of skeletal evolution in european lake and stream stickleback. Evolution 68, 1792–1805, doi: 10.1111/evo.12390 (2014). 24571250

[b17] McDowall R. M. Jordan’s and other ecogeographical rules, and the vertebral number in fishes. J. Biogeo. 35, 501–508, doi: 10.1111/j.1365-2699.2007.01823.x (2008).

[b18] Fowler J. A. Control of vertebral number in teleosts-an embryological problem. Q. Rev. Biol. 45, 148-&, doi: 10.1086/406492 (1970).

[b19] Jordan D. S. Relations of temperature to vertebrae among fishes. Proc. US. Nat. Mus. 14, 107–120 (1892).

[b20] Shikano T. & Merilä J. Body size and the number of vertebrae in the nine-spined stickleback (*Pungitius pungitius*). Biol. J. Linn. Soc. 104, 378–385 (2011).

[b21] Hice L. A., Duffy T. A., Munch S. B. & Conover D. O. Spatial scale and divergent patterns of variation in adapted traits in the ocean. Ecol. Lett. 15, 568–575, doi: 10.1111/j.1461-0248.2012.01769.x (2012). 22462779

[b22] Richardson M. K., Allen S. P., Wright G. M., Raynaud A. & Hanken J. Somite number and vertebrate evolution. Development 125, 151–160 (1998). 9486789 10.1242/dev.125.2.151

[b23] Gomez C. . Control of segment number in vertebrate embryos. Nature 454, 335–339, doi: http://www.nature.com/nature/journal/v454/n7202/suppinfo/nature07020_S1.html (2008). 18563087 10.1038/nature07020

[b24] Lindsey C. C. Factors controlling meristic variation. In Fish Physiology Vol. 11B (eds Hoar D. S. & Randall D. J.) Ch. 3, 197–204 (Academic Press, 1988).

[b25] Head J. J. & Polly P. D. Evolution of the snake body form reveals homoplasy in amniote Hox gene function. Nature 520, 86–89, doi: 10.1038/nature14042 (2015). 25539083

[b26] Wong S. F. L. . Independent regulation of vertebral number and vertebral identity by microRNA-196 paralogs. Proc. Natl. Acad. Sci. USA 112, E4884–E4893, doi: 10.1073/pnas.1512655112 (2015). 26283362 PMC4568285

[b27] Müller J. . Homeotic effects, somitogenesis and the evolution of vertebral numbers in recent and fossil amniotes. Proc. Natl. Acad. Sci. USA 107, 2118–2123, doi: 10.1073/pnas.0912622107 (2010). 20080660 PMC2836685

[b28] Leary R. F., Allendorf F. W. & Knudsen K. L. Inheritance of meristic variation and the evolution of developmental stability in rainbow trout *Salmo gairdneri*. Evolution 39, 308–314 (1985). 10.1111/j.1558-5646.1985.tb05668.x 28564223

[b29] Hubbs C. L. Variations in the number of vertebrae and other meristic characters of fishes correlated with the temperature of water during development. Am. Nat. 56, 360–372, doi: 10.1086/279875 (1922).

[b30] Beacham T. D. & Murray C. B. The effect of spawning time and incubation-temperature on meristic variation in Chum salmon (*Oncorhynchus keta*). Can. J. Zool 64, 45–48, doi: 10.1139/z86-007 (1986).

[b31] Arnold S. J. & Bennett A. F. Behavioral variation in natural-populations. 5. Morphological correlates of locomotion in the garter snake (*Thamnophis radix*). Biol. J. Linn. Soc. 34, 175–190, doi: 10.1111/j.1095-8312.1988.tb01955.x (1988).

[b32] Long J. H. . Testing biomimetic structures in bioinspired robots: How vertebrae control the stiffness of the body and the behavior of fish-like swimmers. Integr. Compar. Biol. 51, 158–175, doi: 10.1093/icb/icr020 (2011). 21576117

[b33] Kelley K. C., Arnold S. J. & Glatstone J. The effects of substrate and vertebral number on locomotion in the garter snake *Thamnophis elegans*. Funct. Ecol. 11, 189–198, doi: 10.1046/j.1365-2435.1997.00077.x (1997).

[b34] Swain D. P. Selective predation for vertebral phenotype in *Gasterosteus aculeatus*-reversal in the direction of selection at different larval sizes. Evolution 46, 998–1013, doi: 10.2307/2409752 (1992). 28564417

[b35] Harding E. F. On the homogeneity of the european eel population (*Anguilla anguilla*). Dana-a Journal of Fisheries and Marine Research 4, 49–66 (1985).

[b36] Lindell L. E. Vertebral number in adders, *Vipera berus*: Direct and indirect effects on growth. Biol. J. Linn. Soc. 59, 69–85, doi: 10.1111/j.1095-8312.1996.tb01453.x (1996).

[b37] Craig J. F. Pike–Biology and Exploitation. (Chapman & Hall, 1996).

[b38] Forsman A. . Pike *Esox lucius* as an emerging model organism for studies in ecology and evolutionary biology: a review. J. Fish Biol. 87, 472–479, doi: 10.1111/jfb.12712 (2015). 26077107 PMC4744780

[b39] Tibblin P. . Evolutionary divergence of adult body size and juvenile growth in sympatric subpopulations of a top predator in aquatic ecosystems. Am. Nat. 168, 98–110, doi: 10.1086/681597 (2015). 26098342

[b40] Larsson P. . Ecology, evolution and management strategies of northern pike populations in the Baltic Sea. AMBIO 44, 451–461, doi: 10.1007/s13280-015-0664-6 (2015). 26022327 PMC4447694

[b41] Tibblin P., Forsman A., Borger T. & Larsson P. Causes and consequences of repeatability, flexibility and individual fine-tuning of migratory timing in pike. J. Anim. Ecol. 85, 136–145, doi: 10.1111/1365-2656.12439 (2016). 26412457

[b42] Berggren H., Nordahl O., Tibblin P., Larsson P. & Forsman A. Testing for local adaptation to spawning habitat in sympatric subpopulations of pike by reciprocal translocation of embryos. PLoS ONE 11(5), e0154488, doi: 10.1371/journal.pone.0154488 (2016). 27139695 10.1371/journal.pone.0154488 PMC4854435

[b43] Webb P. W. Body and fin form and strike tactics of 4 teleost predators attacking fathead minnow (*Pimephales promelas*) prey. Can. J. Fish. Aquat. Sci. 41, 157–165, doi: 10.1139/f84-016 (1984).

[b44] Casselman J. M. Determination of age and growth. In The Biology of Fish Growth (eds Weatherly A. H. & Gill H. S.) Ch. 7, Pages 209–242 (Academic press, 1987).

[b45] Leinonen T., McCairns R. J. S., O’Hara R. B. & Merilä J. Qst-Fst comparisons: evolutionary and ecological insights from genomic heterogeneity. Nature Rev. Genet. 14, 179–190, doi: 10.1038/nrg3395 (2013). 23381120

[b46] Spitze K. Population-structure in *Daphnia obtusa*: quantitative genetic and allozymic variation. Genetics 135, 367–374 (1993). 8244001 10.1093/genetics/135.2.367 PMC1205642

[b47] Merilä J. & Crnokrak P. Comparison of genetic differentiation at marker loci and quantitative traits. J. Evol. Biol. 14, 892–903 (2001).

[b48] Arnold S. J. & Wade M. J. On the measurement of natural and sexual selection: Theory. Evolution 38, 709–719 (1984). 10.1111/j.1558-5646.1984.tb00344.x 28555816

[b49] Gunderson D. R. & Dygert P. H. Reproductive effort as a predictor of natural mortality rate. ICES J. Mar. Sci. 44, 200–209, doi: 10.1093/icesjms/44.2.200 (1988).

[b50] Stearns S. C. The Evolution of Life Histories. (Oxford University Press, 1992).

[b51] Lynch M. & Walsh B. Genetics and Analysis of Quantitative Traits. (Sinauer Associates, Inc., 1998).

[b52] Forsman A. Rethinking phenotypic plasticity and its consequences for individuals, populations and species. Heredity 115, 276–284, doi: 10.1038/hdy.2014.92 (2015). 25293873 PMC4815454

[b53] Stearns S. C. & Koella J. C. The evolution of phenotypic plasticity in life-history traits predictions of reaction norms for age and size at maturity. Evolution 40, 893–913, doi: 10.2307/2408752 (1986). 28556219

[b54] Blob R. W. . Morphological selection in an extreme flow environment: body shape and waterfall-climbing success in the Hawaiian stream fish Sicyopterus stimpsoni. Int. Comp. Biol. 48, 734–749, doi: 10.1093/icb/icn086 (2008). 21669829

[b55] McDowall M. Variation in vertebral number in galaxiid fishes, how fishes swim and a possible reason for pleomerism. Rev. Fish. Biol. Fish. 13, 247–263 (2003).

[b56] Brainerd E. L. & Patek S. N. Vertebral column morphology, C-start curvature, and the evolution of mechanical defenses in tetraodontiform fishes. Copeia, 971–984, doi: 10.2307/1447344 (1998).

[b57] Ackerly K. L. & Ward A. B. How temperature-induced variation in musculoskeletal anatomy affects escape performance and survival of zebrafish (*Danio rerio*). J Exp Zool A Ecol Genet Physiol 325, 25–40, doi: 10.1002/jez.1993 (2016). 26499994

[b58] Swain D. P. & Lindsey C. C. Selective predation for vertebral number of young sticklebacks, Gasterosteus aculeatus. Can. J. Fish. Aquat. Sci. 41, 1231–1233, doi: 10.1139/f84-146 (1984).

[b59] Sasaki K., Fox S. F. & Duvall D. Rapid evolution in the wild: Changes in body size, life-history traits, and behavior in hunted populations of the japanese mamushi snake. Cons. Biol. 23, 93–102, doi: 10.1111/j.1523-1739.2008.01067.x (2009). 18798855

[b60] Reimchen T. E. & Nelson J. S. Habitat and morphological correlates to vertebral number as shown in a teleost, Gasterosteus aculeatus. Copeia 868–874 (1987).

[b61] Hoffman A. A. & Parsons P. A. Evolutionary Genetics and Environmental Stress. (Oxford University Press, 1991).

[b62] Lande R. & Arnold S. J. The measurement of selection on correlated characters. Evolution 37, 1210–1226, doi: 10.2307/2408842 (1983). 28556011

[b63] Orr H. A. Fitness and its role in evolutionary genetics. Nature Rev. Genet. 10, 531–539, doi: 10.1038/nrg2603 (2009). 19546856 PMC2753274

[b64] Kingsolver J. G., Diamond S. E., Siepielski A. M. & Carlson S. M. Synthetic analyses of phenotypic selection in natural populations: lessons, limitations and future directions. Evol. Ecol 26, 1101–1118, doi: 10.1007/s10682-012-9563-5 (2012).

[b65] Roff D. A. The evolution of life histories. (Chapman & Hall, 1992).

[b66] Stearns S. C. Trade-offs in life-history evolution. Funct. Ecol. 3, 259–268, doi: 10.2307/2389364 (1989).

[b67] Swain D. P. Evidence of selection for vertebral number of fry in peamouth, Mylocheilus caurinus. Can. J. Fish. Aquat. Sci. 45, 1279–1290 (1988).

[b68] Frost W. E. & Kipling C. A study of reproduction, early life, weight-length relationship and growth of pike, *Esox lucius L*., in windermere. J. Anim. Ecol. 36, 651–693, doi: 10.2307/2820 (1967).

[b69] Nilsson J., Engstedt O. & Larsson P. Wetlands for northern pike (*Esox lucius L*.) recruitment in the Baltic Sea. Hydrobiologia 721, 145–154, doi: 10.1007/s10750-013-1656-9 (2014).

[b70] Connolly M. H. & Yelick P. C. High-throughput methods for visualizing the teleost skeleton: capturing autofluorescence of alizarin red. J. Appl. Ichty. 26, 274–277, doi: 10.1111/j.1439-0426.2010.01419.x (2010).

[b71] Sokal R. S. & Rohlf F. J. Biometry. (Freeman, 1981).

[b72] O’Hara R. B. & Merila J. Bias and precision in Q(ST) estimates: Problems and some solutions. Genetics 171, 1331–1339, doi: 10.1534/genetics.105.044545 (2005). 16085700 PMC1456852

[b73] Huang L. & Johnson P. The randomization test procedure: testing for a non-zero quadratic effect. in *SAS Conference Proceedings: Midwest SAS Users Group 2004,* Chicago, Illinois. September 26–28, 2004. Available at: http://www.lexjansen.com/mwsug/2004/Statistics/S1_Huang_Johnson.pdf. (Accessed: 25th May 2015).

